# Complete mitochondrial DNA sequence of *Alboglossiphonia lata* Oka, 1910 (Rhynchobdellida: Glossiphoniidae) and its phylogenetic analysis

**DOI:** 10.1080/23802359.2024.2353385

**Published:** 2024-05-17

**Authors:** Panpan Jin, Yu Tian, Erhuan Zang, Lingchao Zeng, Zhaolei Zhang, Jinxin Liu, Linchun Shi

**Affiliations:** a State Key Laboratory for Quality Assurance and Sustainable Use of Dao-di Herbs, Institute of Medicinal Plant Development, Chinese Academy of Medical Sciences & Peking Union Medical College, Beijing, China; b Key Laboratory of Chinese Medicine Resources Conservation, State Administration of Traditional Chinese Medicine of the People’s Republic of China, Engineering Research Center of Chinese Medicine Resource of Ministry of Education, Beijing, China; c Hebei Key Laboratory of Study and Exploitation of Chinese Medicine, Chengde Medical University, Chengde, China

**Keywords:** Mitochondrial genome, *Alboglossiphonia lata*, phylogentic analysis, Glossiphoniidae

## Abstract

The complete mitochondrial genome of *Alboglossiphonia lata* (basionym: *Glossiphonia lata*), sourced from a biodiversity hotspot of China, has been determined and reported in this study. It was 15,236 bp in length and consisted of 13 protein-coding genes, 22 transfer RNA genes, 2 ribosomal RNA genes and three control regions. The mitogenome was deposited GenBank under the accession number PP165800. *A. lata* and other species within the Glossiphoniidae family were clustered together with high bootstrap values. The mitochondrial genome of *A. lata* provides valuable molecular data for further phylogenetic research on the Glossiphoniidae family.

## Introduction

The freshwater leech *Alboglossiphonia lata* belongs to the Rhynchobdellida order and Glossiphoniidae family, and was first reported under the name *Glossiphonia lata* (Oka 1910). This species is commonly found inhabiting aquatic plants and rocks in ponds and marshes, and it can also parasitize in the mantle cavity of freshwater mussels (Siddall et al. 2005). Distributed across various regions, including mainland China, Chinese Taiwan regions, the United States and Japan (Medina Jiménez et al. 2017), *A. lata* is a dorsoventrally flattened leech known for its presence in clean, non-organic polluted streams, irrigation ditches, and open sewers. It primarily feeds on hemolymph of aquatic oligochaetes and snails (Siddall et al. 2005). Members of the Glossiphoniidae family are characterized by their remarkable degree of parental care that captures worms and give them their offsprings carried on their ventral side (Hatto 1968; Kutschera and Wirtz 1986a, 1986b). The fertilized eggs of the Glossiphoniidae family contain a substantial amount of yolk, following the annelid mode of spiral cleavage developmental pattern (Medina Jiménez et al. 2017). Furthermore, *A. lata* serves as a vital model organism in developmental biology research. In 1976, Lukin foresaw the necessity of subdividing the genus *Glossiphonia* and proposed the establishment of a provisional sub-genus named *Alboglossiphonia* (Lukin 1976; Siddall et al. 2005). In 1982, Klemm proposed to raise *Alboglossiphonia* to genus rank (Klemm 1982; Moser et al. 2022). To unravel the genetic characteristics of *A. lata* and elucidate the evolutionary relationship within the Glossiphoniidae family, we conducted sequencing of the complete mitochondrial genome of *A. lata* from a biodiversity hotspot, identifying several new genotypes (Wang et al. 2022).

## Materials and methods

**Figure 1.**
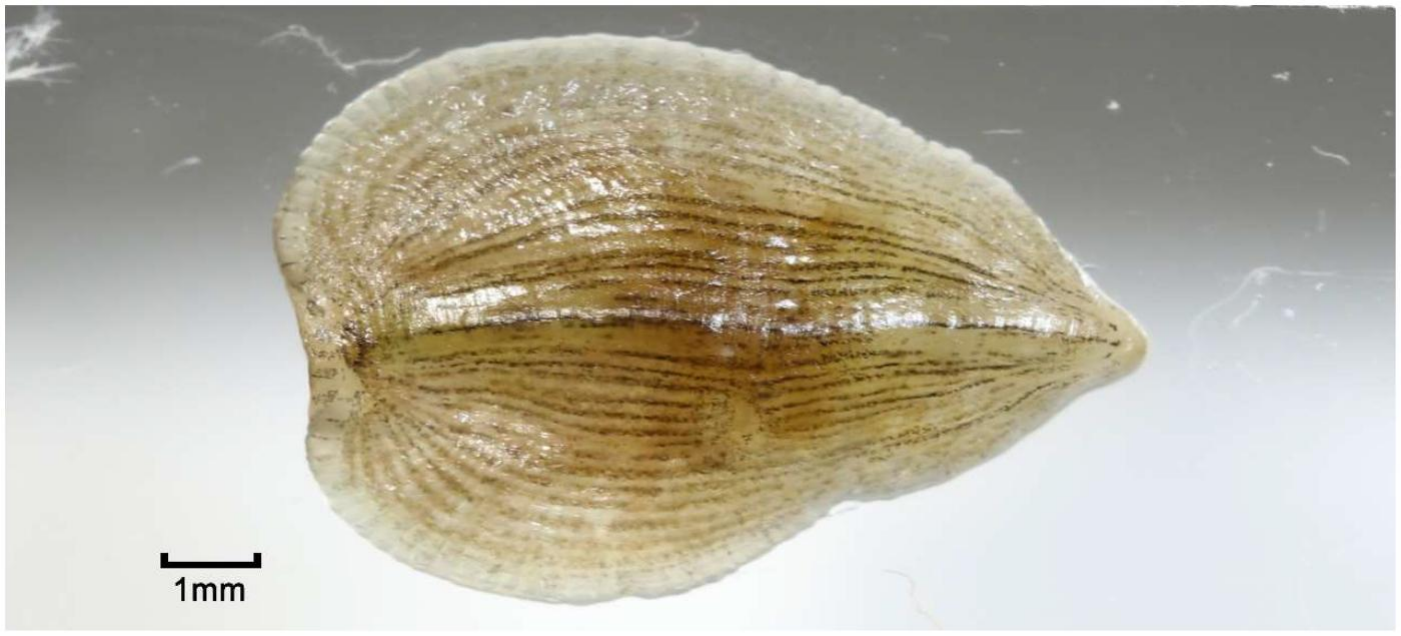
A reference image of *Alboglossiphonia lata* sequenced in this study. The specimens were collected in Chengde City, Hebei Province, China (coordinates: N 41*°*03’, E117*°*57’). The photograph was taken by Linchun Shi at the Institute of Medicinal Plant Development.

Specimen samples (Figure 1) were collected from Chengde City, Hebei Province, China (N 41*°*03′, E117*°*57′). The specimens were deposited at the Institute of Medicinal Plant Development (Linchun Shi, lcshi@implad.ac.cn) under the voucher number HSLT0014. Genomic DNA extraction from muscle samples was performed using the TIANamp Genomic DNA Kit (Tiangen, Beijing, China) following the manufacturer’s protocol. The DNA yield and purity were assessed using a NanoDrop 2000 ultra-micro spectrophotometer (Thermo Scientific, USA) and quantified with the Qubit 4.0 (Thermo Scientific, USA). Following genomic DNA extraction, PCR-free libraries were generated, and subsequent sequencing was conducted on the Illumina NovaSeq platform with PE150 model. A total of 3.5 Gb pair-end raw data were generated and then Trimmomatic v0.38 (Bolger et al. 2014) was employed to filter low-quality reads and remove sequencing adapters. The complete circular mitochondrial genome was executed using GetOrganelle v1.7.7.0 (Jin et al. 2020), and MITOS (Bernt et al. 2013) was applied for mitochondrial genome annotation. Manual corrections were undertaken through annelid multiple sequence alignment to enhance the precision of the mitochondrial genome. OrganellarGenomeDRAW (OGDRAW) version 1.3.1 (Greiner et al. 2019) was utilized to draw the circular map of the mitochondrial genome. The phylogenetic position of *A. lata* was confirmed using RAxML v8.0.0 (Stamatakis 2014) to create a Maximum Likelihood (ML) tree.

## Results

**Figure 2.**
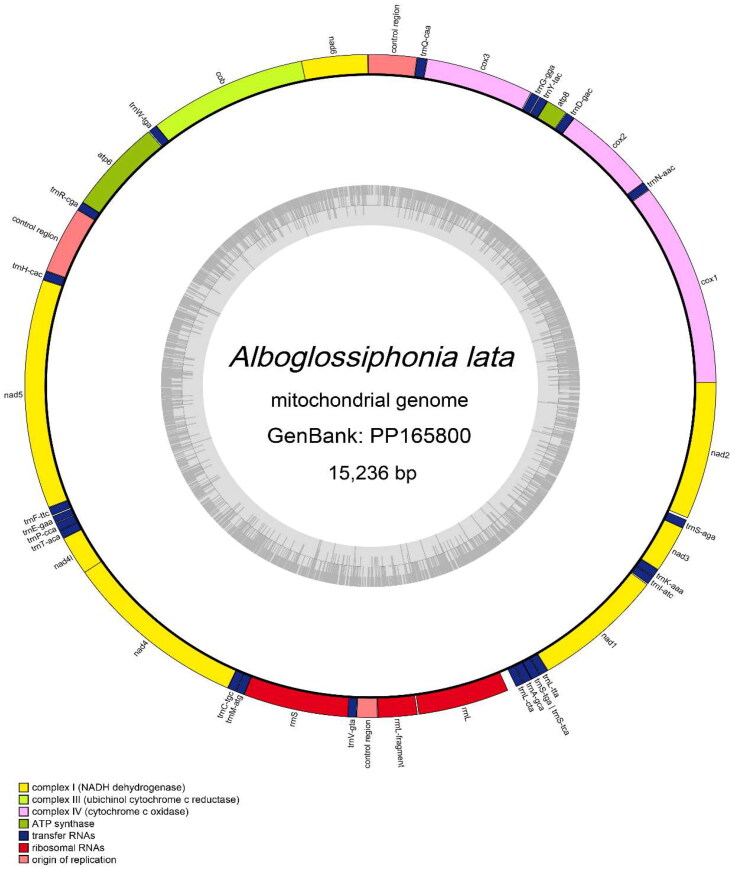
Circular map of the *Alboglossiphonia lata* mitochondrial genome (GenBank accession: PP165800), including 13 protein-coding genes, 22 tRNAs, 2 rRNAs and three control regions.

The complete mitochondrial genome of *A. lata* formed a circular molecule measuring 15,236 bp in length, with a coverage depth of 748.07X (Supplementary Figure S1). This genome was deposited in GenBank under accession number PP165800. The nucleotide base content of *A. lata* mitogenome was 35.9% A, 39.3% T, 13.1% C, and 11.7% G. It consisted of 13 protein-coding genes (PCGs), 22 tRNA genes, 2 rRNA genes, and three control regions (Figure 2). The length of the PCGs ranged from 159 bp (*atp8*) to 1702 bp (*nad5*). All protein-coding genes used the typical ATN as the start codon (ATT: ATA: ATG= 2:1:10), except for *cox3* and *nad2* with ATT as the start codon, *atp6* with ATA as the start codon, and other protein-coding genes use ATG as the start codon. Most PCGs terminated with the TAA codon, with the exceptions of *cox3*, *nad5*, and *nad3*. The lengths of 22 tRNA genes ranged from 55 bp (*trnE*) to 69 bp (*trnQ*), and all produced the expected typical cloverleaf structures.

**Figure 3.**
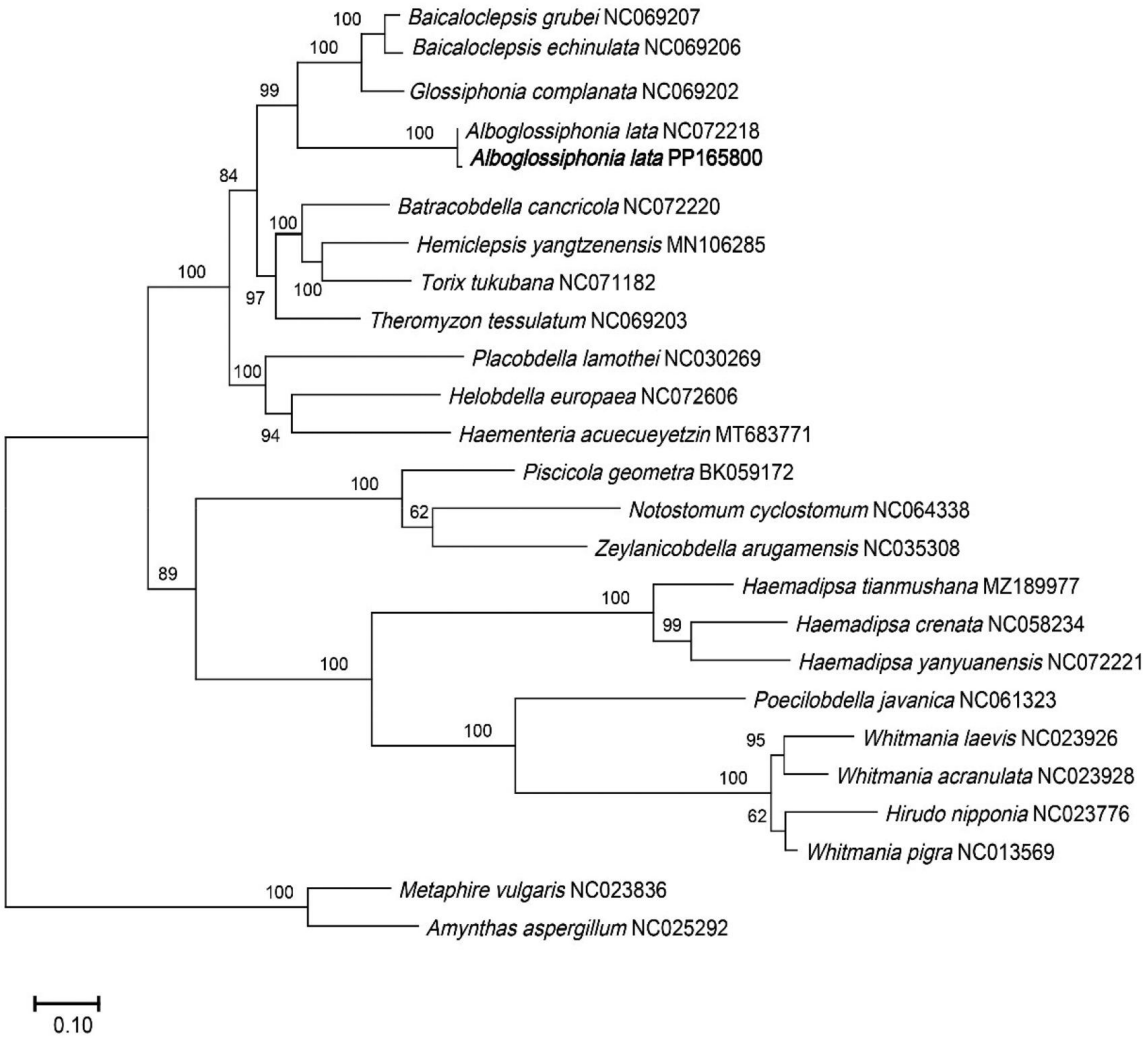
The phylogenetic position for *Alboglossiphonia lata* according to the ML phylogenetic tree. The bootstrap support values were shown on the branches. Bold text denotes species of which the sequences were newly revealed in this study. The amino acid sequences of the 13 PCGs of following species were also used: *B. grubei* NC069207 (Bolbat et al. 2022), *B. echinulata* NC069206 (Bolbat et al. 2022), *G. complanata* NC069202 (Bolbat et al. 2022), *A. lata* NC072218 (unpublished), *B. cancricola* NC072220 (unpublished), *H. yangtzenensis* MN106285 (Xu et al. 2021), *T. tukubana* NC071182 (unpublished), *T. tessulatum* NC069203 (Bolbat et al. 2022), *P. lamothei* NC030269 (Oceguera-Figueroa et al. 2016), *H. europaea* NC072606 (Rashni et al.^2023^), *H. acuecueyetzin* MT683771 (Sosa-Jiménez et al. 2020), *P. geometra* BK059172 (Bolbat et al. 2021), *N. cyclostomum* NC064338 (Direct Submission), *Z. arugamensis* NC035308 (Direct Submission), *H. tianmushana* MZ189977 (Lu et al. 2022), *H. crenata* (Wang et al. 2022), *H. yanyuanensis* NC072221 (unpublished), *P. javanica* NC061323 (unpublished), *W. laevis* NC023926 (unpublished), *W. acranulata* NC023928 (unpublished), *H. nipponia* NC023776 (Xu et al. 2016), *W. pigra* NC013569 (unpublished), *M. vulgaris* NC023836 (Zhang et al. 2016), *A. aspergillus* NC025292 (Zhang et al. 2016).

To conduct the phylogenetic analyses, the complete mitochondrial genome sequences of 24 Clitellata species were retrieved from the National Center for Biotechnology Information (NCBI). *M. vulgaris* (NC023836) and *A. aspergillum* (NC025292) were used as outgroups. The phylogenetic results indicated that all the species belonging to Glossiphoniidae family were clustered together. Compared with *A. lata*, *G. complanate*, *B. grubei*, and *B. echinulate* demonstrated a closer evolutionary connection. Our newly reported specimen exhibited the closest relationship with the previously released *A. lata* (NC072218), supported by a robust bootstrap value of 100 (Figure 3).

In this study, we conducted a comparative analysis between *A. lata* (NC072218) and *A. lata* (PP165800), revealing a total of 303 single nucleotide polymorphisms (SNPs) (Table 1). Notably, a significant proportion of these SNPs (241) were localized within protein-coding regions. Among the 13 protein-coding genes (PCGs) examined, *nad5* exhibited the highest variability, containing 34 SNPs, followed closely by the *cox1* and *cob* genes, with 32 and 30 SNPs, respectively. Furthermore, our analysis detected 37 positions within the PCGs where the encoded amino acid types diverged between the two *A. lata* strains.

**Table 1.** The number of SNPs and amino acid differences in the 13 protein-coding genes between mitochondrial genomes of *A. lata* (NC072218, as reference) and *A. lata* (PP165800).

	13 protein-coding genes												
	*cox1*	*cox2*	*atp8*	*cox3*	*nad6*	*cob*	*atp6*	*nad5*	*nad4l*	*nad4*	*nad1*	*nad3*	*nad2*
SNPs	32	14	2	18	12	30	17	34	6	28	15	13	20
Amino acid difference	1	2	0	3	2	5	3	5	2	5	1	5	3

## Discussion and conclusion

In this study, we assembled the mitochondrial genome sequence of *Alboglossiphonia lata*, previously named as *Glossiphonia lata*. Phylogenetic analyses revealed that *A. lata* and other species of Glossiphoniidae family were clustered together with a high bootstrap value. Although two sequences of *A. lata* were clustered in a clade, there were still a significant number of SNPs in the protein-coding regions between the mitochondrial genomes. The PCGs in the mitochondrial genomes of the two *A. lata* appeared to encode proteins with substantial amino acid differences. These genetic divergences likely represent instances of allopatric speciation, attributed to the segregation within the species’ distribution ranges. This study provides valuable molecular data, contributing to a deeper understanding of the evolutionary status of *A. lata* and the phylogeny of the Glossiphoniidae family.

## Supplementary Material

Supplemental Material

## Data Availability

The genome sequence data that support the findings of this study are openly available in GenBank of NCBI at [https://www.ncbi.nlm.nih.gov] (https://www.ncbi.nlm.nih.gov/) under the accession no. PP165800. The associated Bio-Project, Bio-Sample and SRA numbers are PRJNA1067814, SAMN39557300 and SRR27748508.
