# Supplementary figures and images for: Complete mitochondrial DNA sequence of Alboglossiphonia lata Oka, 1910 (Rhynchobdellida: Glossiphoniidae) and its phylogenetic analysis
Source: Mitochondrial DNA B Resour. 2024 May 17;9(5):652–6. doi: 10.1080/23802359.2024.2353385 (PMC11104703; doi:10.1080/23802359.2024.2353385)

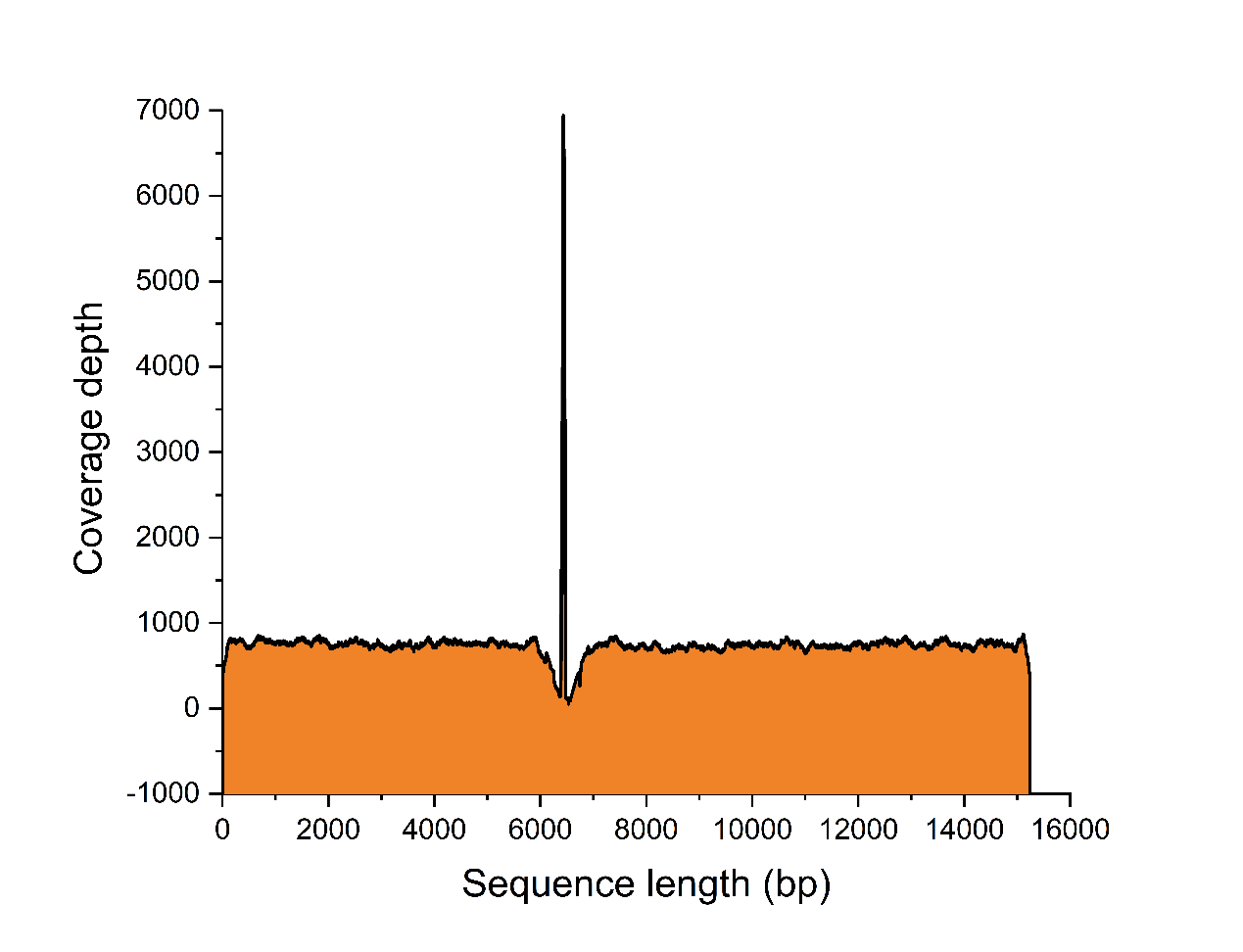

Supplement: Supplemental Material [file TMDN_A_2353385_SM5467.tif]
